# Evolving prevalence of haematological malignancies in orphan designation procedures in the European Union

**DOI:** 10.1186/s13023-017-0567-7

**Published:** 2017-01-21

**Authors:** Benedetta Polsinelli, Stelios Tsigkos, Frauke Naumann-Winter, Segundo Mariz, Bruno Sepodes

**Affiliations:** 1 European Medicines Agency, 30 Churchill Place Canary Wharf, London, E14 5EU UK; 2 Bundesinstitut für Arzneimittel und Medizinprodukte, Kurt-Georg-Kiesinger-Allee 3, 53175 Bonn, Germany; 3 Universidade de Lisboa, Faculdade de Farmácia, Avenida Professor Gama Pinto, 1649-003 Lisboa, Portugal

**Keywords:** European orphan regulation, Committee for orphan medicinal products, Orphan designation, Haematological malignancies, Prevalence, Market exclusivity

## Abstract

The Committee for Orphan Medicinal Products (COMP) evaluates prevalence of rare conditions as one of the criteria for granting an orphan designation with a prevalence threshold of 5 in 10.000. At the time of Marketing Authorisation (MA) these criteria are reassessed to ensure they are still met. The COMP has noted discordance between the prevalence of certain haematological malignancies at the time of Orphan Designation and at the time of Marketing Authorisation. Consequently, we conducted a retrospective assessment of Chronic Lymphocytic Lymphoma and Multiple Myeloma/Plasma cell Myeloma as well as several other haematological rare aetiologies frequently subject of orphan designation. These were: Diffuse large B-Cell Lymphoma (DLBCL), Follicular Lymphoma (FL), Cutaneous T-Cell Lymphoma (CTCL), Mantle Cell Lymphoma (MCL) and Chronic Myeloid Leukaemia (CML). The review used submissions as well as recent publications and results from external and EMA databases. As a first step in the analysis, an increase over time in the number of people affected was evident for four conditions in the COMP designation documents, whereas for DLBCL, FL, CTCL and MCL there had been no significant change, since the introduction of the Regulation in 2000. Specifically, the prevalence estimates increased from 1.2 to 3.6 per 10,000 for multiple myeloma, from 0.4 to 1.7 in acute lymphoblastic leukaemia, and from 2.7 to 4.85 for chronic lymphocytic leukaemia/small lymphocytic leukaemia and 1 to 2 in 10,000 for chronic myeloid leukaemia. The reasons for the changes in the prevalence of these four haematological conditions over the last 15 years were not assessed but recent publications have alluded to better outcomes due to new treatments being made available. In addition, many orphan diseases have a median age of onset over 60 years so that also the aging of the population may be a relevant contributing factor.

## Background

In 2000 the European Union introduced legislation to encourage the development of medicinal products for rare diseases. This legislation established a scientific Committee including patients as full members, the Committee for Orphan Medicinal Products (COMP) whose role is to assess submissions for the designation of medicinal products intended for the treatment, diagnosis or prevention of a medical condition which is considered rare. The COMP meets every month and discusses submissions for Orphan Designation. The main criteria for assessment are the eligibility of a proposed medical condition as a distinct medical entity, the medical plausibility that the proposed medicinal product holds therapeutic promise in the proposed condition, that the prevalence of the condition is less than or equal to 5 in 10,000 and where authorised medicinal products exist in Europe, the medicinal product will bring significant benefit for the patients affected by the condition. The outcome of the positive assessments (positive opinions) are made public each month following each plenary, and can be found on a dedicated Orphan Designation database available on the European Medicines Agency and European Commission websites.

**Fig. 1:**
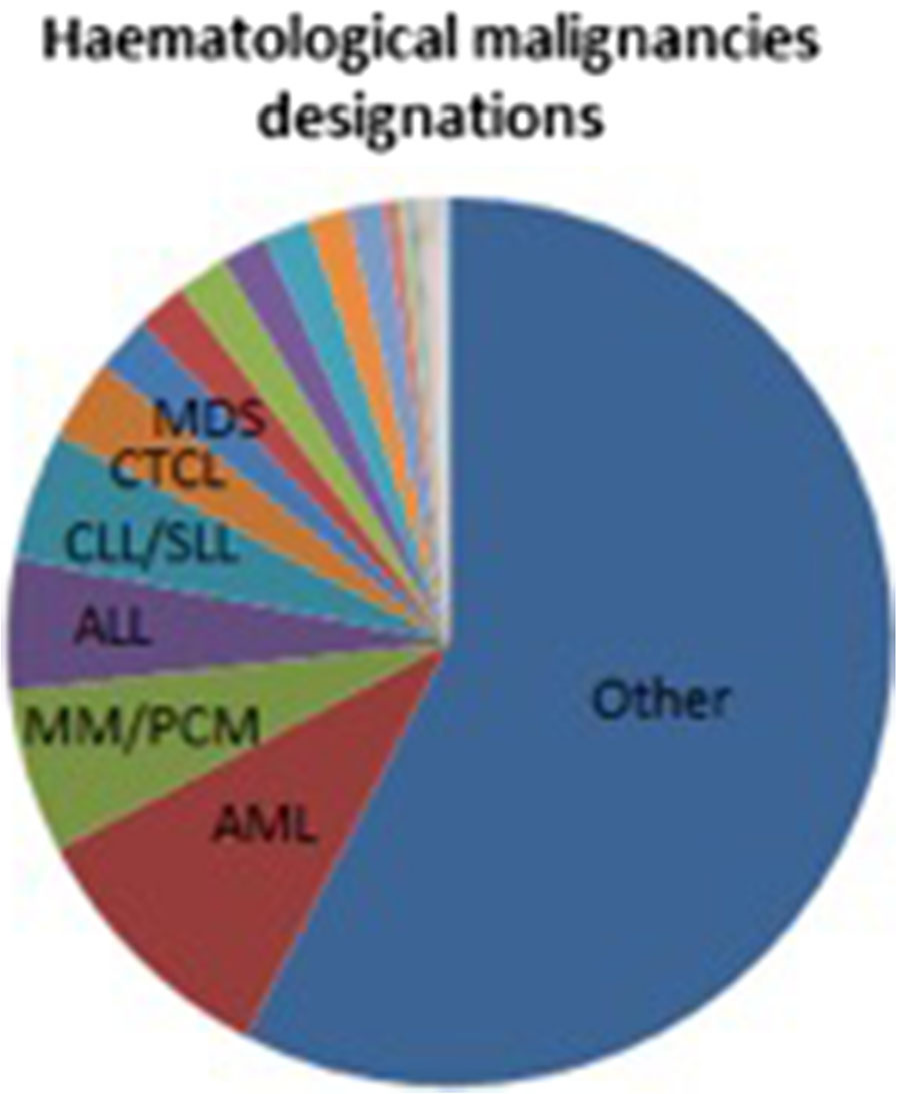
Distribution of all orphan designations for haematological malignancies in oncology 2000–2015 (Percentage details: AML 9,6%; MM 6%; ALL 5%; CLL/SLL 5%; CDCL 3%; MDS 2%)

The COMP receives many submissions for oncological conditions, around 35% of all submissions each year. Rare haematological malignancies (HMs) represent approximately 42% (228 OD in HMs/538 total ODs) of designations of orphan medicinal products in oncology granted by the COMP from 2000 until May 2015 (Fig. 1).

Recently, members of the COMP have noted discordance between prevalence calculations submitted for some of these conditions and what is reported in the literature in multiple myeloma and chronic lymphocytic leukaemia/small lymphocytic lymphoma. This led to the sponsor’s being requested during the procedure to re-calculate the prevalence, or justify in an oral explanation the basis for their calculation. As a result the EMA COMP and the Orphan Medicines Office conducted a retrospective analysis of the most frequent haematologic conditions submitted for Orphan Designation and compared the prevalence reported by sponsors to those reported in recent publications for each of the conditions. The WHO 2008 Classification system of haematological malignancies has been used where possible by the COMP for haematological conditions and was the basis of the conditions selected for this analysis. The conditions chosen for the assessment were: acute myeloid leukaemia (AML), acute lymphocytic leukaemia, (ALL), cutaneous T-cell lymphoma (CTCL), diffuse large B-cell lymphoma (DLBCL), follicular lymphoma (FL), Chronic myeloid leukaemia (CML), chronic lymphocytic leukaemia/small lymphocytic lymphoma (CLL/SLL), mantle-cell lymphoma (MCL), Multiple Myeloma (MM), Hodgkin’s Lymphoma (HL) and myelodysplastic syndrome (MDS).

The retrospective analysis showed that there was an increase in the prevalence of four conditions namely multiple myeloma, acute lymphocytic leukaemia, chronic lymphocytic leukaemia/small lymphocytic lymphoma and chronic myeloid leukaemia. In the case of multiple myeloma and chronic lymphocytic leukaemia an increase was noted at assessment by the COMP and not the submissions from the sponsors. The reasons for the change in the prevalence in these conditions were not the primary aim of the analysis, however it was noted that peer reviewed publications of the management and treatment of patients affected by these conditions have reported better outcomes potentially linked to the use of new medicinal products introduced over that last 10 years.

## Methodology

We looked at positive orphan designations in the EMA database (at the time of initial Orphan Designation and at the time of maintenance of the Orphan Designation) for the following haematological conditions most commonly submitted to the COMP: acute myeloid leukaemia (AML), acute lymphoblastic leukaemia, (ALL), cutaneous T-cell lymphoma (CTCL), diffuse large B-cell lymphoma (DLBCL), follicular lymphoma (FL), Chronic myeloid leukaemia (CML), chronic lymphocytic leukaemia/small lymphocytic lymphoma (CLL/SLL), mantle-cell lymphoma (MCL), multiple myeloma (MM), Hodgkin’s lymphoma (HL) and myelodysplastic syndrome (MDS). We examined retrospectively the prevalence estimates across time (from 2000 to 2015) in these designations. The criteria applied to choose the conditions to be analysed were the number of initial positive orphan designations granted and number of positive maintenance of Orphan Designations (this step is conducted at the time of Marketing Authorisation application, 3 to 4 years after the designation on average for all designations), as identified on the EMA website [2]. Submissions for MA that were withdrawn following COMP negative opinion were not included in the analysis. The COMP public summary of opinion reports which appear on the EMA website and the minutes of the COMP meetings have been used to identify the prevalence estimates provided by the sponsors, the type of prevalence proposed by the sponsors (point prevalence or partial prevalence) and any question related to the sponsor’s prevalence calculation raised by the Committee. Partial prevalence estimates are reported over a limited period of time, such as 5-year or 10-year prevalence, capturing all living patients who had been diagnosed cases in the timespan of the preceding 5 or 10 years. Point prevalence is the proportion of a population that has the condition at a specific point in time irrespective of the time of diagnosis. A literature search using Pubmed was conducted to identify recent prevalence calculations for these conditions and epidemiological databases (Haematological Malignancy Research Network, Cancer research UK, National Cancer Data Repository) were used to compare with the outcomes of the COMP evaluation [3, 4, 8]. It has been noted that there is little variance in reporting rates between Member States for these conditions.

### Limitations of the methods

This is a retrospective study based on the analysis of data gathered from the scientific documents (applications) submitted over time. The prevalence estimates reported result from the calculation exercise performed by each applicant, whose methodology can vary individually, despite the European Medicines Agency guidance document, “Point to consider on the calculation and reporting of the prevalence of a condition for orphan designation”; this document has however not been updated since 2002. Furthermore, the prevalence values have not been standardised, meaning that they have been pulled together irrespective of epidemiological index used. Therefore, variability of data due to methodological factors needs to be taken into account in the interpretation of the findings. In many ways this is main driver of the variance. Factors such as the use or not of European Member State cancer registries as well as the selection of publications based on older or current articles can drive variance has been noted at the time of assessment.

Another point worth to clarifying is related to the type of prevalence reported. In earlier designations for multiple myeloma and chronic lymphocytic leukaemia/ small lymphocytic lymphoma, partial prevalence was accepted as life expectancy was considered to be limited. As the literature reported improvements in survival and in view of the aging population, COMP members have recently requested point prevalence as it also helps to bring into consideration the increase in the reported prevalence for the two conditions of multiple myeloma and chronic lymphocytic leukaemia/ small lymphocytic lymphoma. This was not the case for ALL. For the other conditions studied, the COMP did not find any increase in the literature of either point or partial prevalence. It should however be noted that there was an increase in prevalence in CML with the introduction of Glivec [9].

## Results

**Fig. 2:**
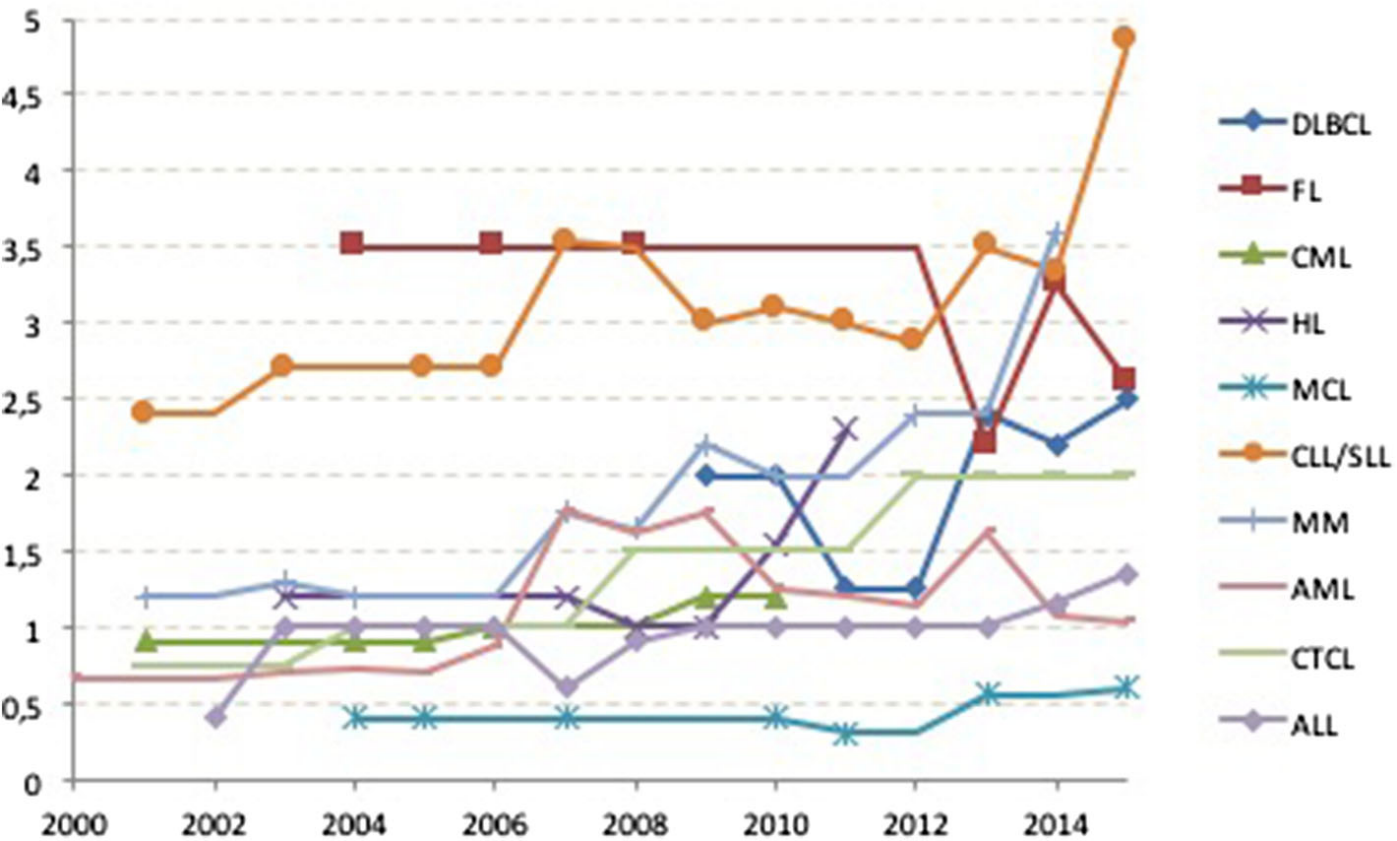
Prevalence reported for OD trends submitted to the COMP for Diffuse Large B-Cell Lymphoma (DLBCL), Follicular Lymphoma (FL), Chronic Myeloid Leukaemia (CML), Hodgkin’s Lymphoma (HL), Mantle Cell Lymphoma (MCL), Chronic Lymphocytic Leukaemia/ Small Lymphocytic Lymphoma CLL/SLL, Multiple Myeloma (MM) and Acute Myeloid Leukaemia (AML) and Acute Lymphoblastic Leukaemia (ALL) between 2000 and 2015

Figure 2 indicates the prevalence reported for the haematological conditions analysed.

**Fig. 3:**
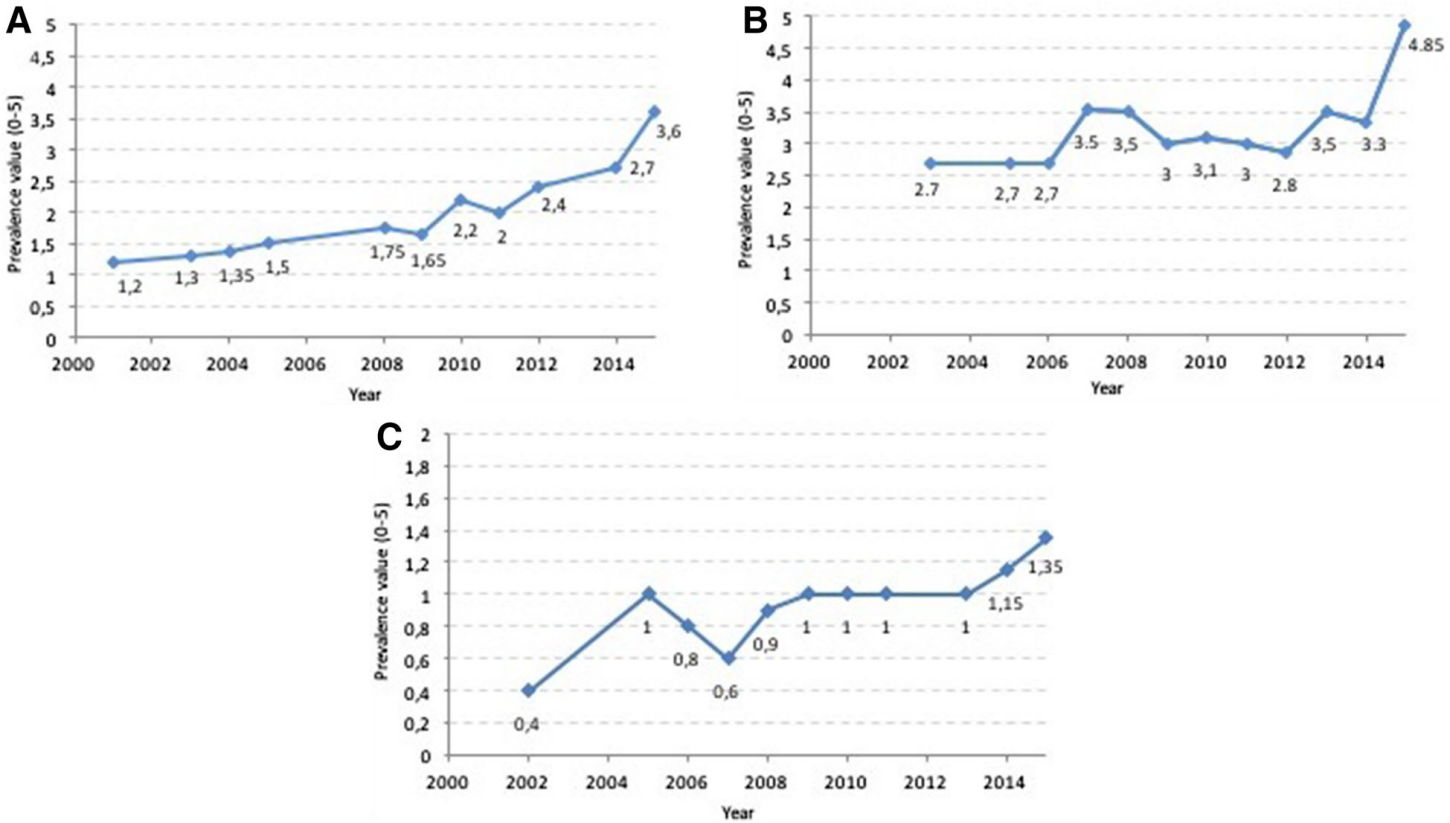
Prevalence trends as reported above submitted to the COMP for **a**) multiple myeloma, **b**) chronic lymphocytic leukaemia/small lymphocytic lymphoma and **c**) acute lymphoblastic leukaemia during the timeframe 2000–2015

As can be seen reporting rates have been stable for DLBCL, FL, HL, MCL and AML and the published literature does not report changes. There has been an increase in CML from 1 in 10,000 reported in 2001, to 2 in 10,000 in 2012. A more significant rise in CLL/SLL and MM can be seen in this report from 2013 for MM and 2014 for CLL/SLL. In the case of ALL, there has been a slow increase from 0.4 in 10,000 in 2001 to 1.35 in 10,000 in 2015. This is presented in the Fig. 3.

For conditions of very low prevalence, chronic myeloid leukaemia and acute lymphoblastic leukaemia the COMP is not requesting changes in how the prevalence calculations are made for orphan submissions. It has only been in conditions where there have been public reports of higher prevalence that the COMP has become more demanding regarding the prevalence calculations submitted.

In the conditions where there was an increase in the prevalence since the introduction of the orphan legislation, 7 medicinal products have been authorised, for multiple myeloma (Carfilzomib, Daratumumab, Dexamethasone, Lenalidomide, Panobinostat, Pomalidomide, and Thalidomide), 3 for chronic lymphocytic leukaemia/ small lymphocytic lymphoma (Obinutuzumab and Ofatumumab, ibrutinib), 6 for acute lymphoblastic leukaemia (Clofarabine, Dasatinib, Imatinib, Mercaptopurine, Nelarabine, Ponatinib) and 5 for chronic myeloid leukaemia (Bosutinib, Dasatinib, Imatinib, Nilotinib, and Ponatinib).

One medicinal product each was authorised under the orphan legislation for follicular lymphoma (Gazyvaro), two for mantle cell lymphoma (Temsirolimus, Ibrutinib), one for hodgkin’s lymphoma (Brentuximab Vedotin), and four for acute myeloid leukaemia (Ceplene, Azacidine, Decitabine and Histamine dihydrochloride). There have been none authorised for diffuse large B-cell lymphoma. It should also be noted that some products have received authorisation for these conditions without Orphan Designation such as idelalisib.

From the data available to the EMA, it is not possible to conclude as to whether the introduction of these medicines has improved patient outcomes.

## Discussion

The COMP is mandated to give access to incentives for the development of medicinal products and thereby make medicinal products available for patients affected by rare diseases. According to the regulation 141/2000, the threshold of prevalence of a proposed orphan condition has to be met “at the time of application”. Since the designation is reviewed at MA, updated prevalence figures may be necessary at review. In reviewing medicines intended for rare diseases, the Committee noted discrepancies in the reporting of current prevalences by sponsors in their submissions for certain haematological conditions in Orphan Designation submissions both at the time of initial designation and at the time of marketing authorisation review (so-called maintenance of Orphan Designation) compared with recent published reviews. Prevalence changes can be expected in case of rising incidence or improved survival of patients with the condition. Expected changes in the demography of the European the population (aging of the babyboomer generation) are expected to result in substantial increase of the prevalence of conditions diseases with a median onset of over 60 years.

There has been some speculation within the COMP and in the published literature regarding the number of new products introduced in the market since it started designating medicinal products and their impact on improvement in patient outcome. Whether outcome and survival improvements are the main factors responsible for increased prevalence is open up for interpretation, as most of the conditions have had at least one product authorised for use in the target condition, except diffuse large B-cell Lymphoma.

We now present an example for each of the conditions where increased prevalence has been observed.

### Multiple myeloma

The minutes of the assessment of EMA/COMP Procedure EMA/OD/277/14 for multiple myeloma highlight the first case where the COMP questioned the prevalence calculation submitted by a sponsor for this condition [5]. The sponsor was asked to re-evaluate the prevalence based on more recent epidemiological publications and consultation of databases, which have shown improved survival and prognosis. The sponsor’s answer acknowledged the recently reported improved prognosis and patient survival for the condition compared to older publications (6.1 years survival reported in 2015 versus 4.5 years in older publications). Results from literature search show an improved survival for multiple myeloma has been recently reported in the literature [6]. Epidemiological analysis published by Kumar and Sant groups (2015) provides evidence in support of continued improvement in the survival outcomes within the past decade.

Interestingly, significant survival improvement has been seen among the older patients, whereas previously described improvements were mainly in the younger patients, possibly as a reflection of lower utilisation of stem cell transplantation and reduced access to clinical trials evaluating new drugs [1, 7].

Databases such as Cancer research UK indicate similar results to recent publications with improved current survival rates across several European countries: the 5-year survival rates range from 23.1% to 46.7% [10, 11]. This database includes myeloma survival rates in the UK since the early 1970s [8]: over this period, five-year age-standardised net survival for adults increased from 11.9% in the ‘70s to 47% in 2010–2011. The 10-year survival rate increased from 6.4% to 32.5% in the same timeframe. These findings put into question the usefulness of the 5-year point prevalence for the purpose of orphan designation, and the COMP now requires that point prevalence be used for this condition.

### Chronic Lymphocytic leukaemia/ small lymphocytic lymphoma

The minutes of the assessment of EMA/COMP Procedure EMA/OD/108/15 [5] show that the sponsor was asked to re-assess prevalence based on possible increasing survival rates reported in the literature [1]. The sponsor’s response acknowledged the increase and amended the prevalence estimate to 4.9 per 10.000. The findings in the literature are also supported by data in the Cancer Research UK and the National Cancer Data Repository- NCDR) [8].

## Conclusion

The COMP recently noted a discrepancy in prevalence calculations and what has been reported in publications for some haematological conditions, which qualify as rare conditions under the European Orphan legislation.

Sponsors are reminded that previously accepted prevalence calculations which are publicly accessible on the EMA website, are indicative of the prevalence at the moment that the designation was made. At the time of Maintenance of Orphan Status at the time of Marketing Authorisation, the sponsors should check current reported prevalences as they can change over time due to a multitude of factors such as the introduction of new medicinal products and an improved understanding of the condition. The COMP actively and critically assesses the prevalence submissions and considers up to date epidemiological literature and database reports in a given condition. Prevalence calculations, especially for extremely rare diseases are typically associated with a high degree of uncertainty. Therefore, justifications of selection of sources or sensitivity analyses are recommended. Criteria for prevalence calculations, such as the acceptability of partial prevalence versus point prevalence, can change in case of reported improvements in patient outcomes and additionally may depend on characteristics of the disease. Therefore, sponsors are asked to specify and justify the epidemiological index on which they base their final conclusion of prevalence.

The impact of the new medicinal products authorised under the European Orphan Legislation as well as those not authorised under this incentive programme, on patient outcomes and survival was not the primary aim of this retrospective analysis and would require further data and assessment. It was primarily the rising concern in the COMP of the discrepancy seen between reported submitted prevalence’s for some of these conditions and what is being currently reported in the public domain which was a primary motive for this retrospective analysis.

## Abbreviations

ALL: Acute lymphocytic leukaemia
CLL/SLL: Chronic lymphocytic leukaemia/ small lymphocytic lymphoma
COMP: Committee of orphan medicinal products
EMA: European Medicines Agency
EU: European Union
HMRN: Haematological Malignancy Research Network
HMs: Haematological malignancies
MM: Multiple myeloma
NCDR: National Cancer Data Repository
OD: Orphan designation
OMPs: Orphan medicinal products
WHO: World Health Organization
